# *Prevotella* in Pigs: The Positive and Negative Associations with Production and Health

**DOI:** 10.3390/microorganisms8101584

**Published:** 2020-10-14

**Authors:** Samat Amat, Hannah Lantz, Peris M. Munyaka, Benjamin P. Willing

**Affiliations:** 1 Department of Agricultural, Food and Nutritional Science, University of Alberta, Edmonton, AB T6G 2P5, Canada; samat.amat@ndsu.edu (S.A.); lantz@ualberta.ca (H.L.); munyakam@ualberta.ca (P.M.M.); 2 Department of Microbiological Sciences, North Dakota State University, Fargo, ND 58108-6050, USA

**Keywords:** animal production and health, gut microbial ecology, immune system, microbiota, *Prevotella*, pig

## Abstract

A diverse and dynamic microbial community (known as microbiota) resides within the pig gastrointestinal tract (GIT). The microbiota contributes to host health and performance by mediating nutrient metabolism, stimulating the immune system, and providing colonization resistance against pathogens. Manipulation of gut microbiota to enhance growth performance and disease resilience in pigs has recently become an active area of research in an era defined by increasing scrutiny of antimicrobial use in swine production. In order to develop microbiota-targeted strategies, or to identify potential next-generation probiotic strains originating from the endogenous members of GIT microbiota in pigs, it is necessary to understand the role of key commensal members in host health. Many, though not all, correlative studies have associated members of the genus *Prevotella* with positive outcomes in pig production, including growth performance and immune response; therefore, a comprehensive review of the genus in the context of pig production is needed. In the present review, we summarize the current state of knowledge about the genus *Prevotella* in the intestinal microbial community of pigs, including relevant information from other animal species that provide mechanistic insights, and identify gaps in knowledge that must be addressed before development of *Prevotella* species as next-generation probiotics can be supported.

## 1. Introduction

A diverse and dynamic microbial community (known as microbiota) resides within the pig gastrointestinal tract (GIT) and contributes to host health and performance by mediating nutrient metabolism, modulating the immune system, and providing colonization resistance against pathogens [1,2,3]. These beneficial traits of the gut microbiota are influenced by host condition, dietary, environmental and management factors. Perturbation of well-balanced (homeostatic) microbial populations is known to have long-term adverse effects on host physiology [4,5,6,7]. For example, antibiotic perturbation of the piglet GIT microbiota in early life has been shown to be associated with impaired gut microbiome development and altered metabolic regulation later in life [8]. Furthermore, compromised resistance against infectious agents has been reported in pigs raised under more hygienic and controlled conditions [9]. Pigs that develop post-weaning diarrhea have also been observed to have distinct early-life microbiota profiles [10]. Restoration of the gut microbiota homeostasis in pigs has become an active area of research in order to enhance animal health and production through different strategies. For example, fecal microbiota transplantation (FMT) is one of the strategies used to improve pig health through modulation of the gut microbiota [11,12,13]. Understanding the biology and role of key members of the pig gut microbial community in defining host physiology is necessary to deduce the complex guiding principles that govern microbiota–host interactions in the gut, which will ultimately facilitate the development of gut microbiota-targeted strategies to improve pig health and productivity.

*Prevotella* is one of the most predominant genera across the large intestine of pigs [14,15]. This genus is also a central constituent in one of the two most common bacterial enterotypes of gut microbiota in pigs, especially after weaning (*Prevotella* vs. *Treponema*-dominant enterotypes, *Prevotella* vs. *Ruminococcaceae* enterotypes) [16,17,18,19]. The *Prevotella*-driven enterotype has shown positive associations with animal traits including feed intake [18], feed efficiency [17], weight gain [19] and incidence of diarrhea [10], suggesting that *Prevotella* is important in mediating growth performance and disease resilience in pigs. Moreover, network analysis revealed that *Prevotella* is a highly connected taxa that exhibits strong competitive and cooperative interactions with many members of gut microbiota [12,17]. Considering the new definition of keystone taxa [20], *Prevotella* can be considered as a keystone taxon, as it has a profound influence on community structure and function of the gut microbiota in pigs.

*Prevotella* spp. are not only key members of the intestinal microbiota in pigs, but are commonly present in many different mammalian species including humans [21], non-human primates [22], mice [23], ruminants [24] and poultry [25]. Being one of the most predominant genera among the rumen microbiota, *Prevotella* spp. contributes significantly to both carbohydrate and nitrogen metabolism in ruminants [26,27,28]. Human gut *Prevotella* has received considerable attention due to its associations with host diet and metabolism [21,29], immune response [30], and mental health [31]. The role of *Prevotella* in defining human health, however, still remains controversial [32]. Many studies have employed rodent models to study *Prevotella* in humans [33,34,35], however, given the high degree of similarity in anatomy, physiology and immunology between pigs and humans, pig models may be more clinically relevant to humans than rodent models [36,37,38]. Therefore, providing a cohesive summary of the findings on gut *Prevotella* from the studies conducted in pigs may also enhance our understanding of the role of *Prevotella* in human health. The overall goal of this review is to summarize the current knowledge of *Prevotella* in the pig gut and its associations with microbiome-mediated growth performance and host health. In addition, some challenges associated with characterization of *Prevotella* in the pig gut, and future research directions to overcome these challenges will be discussed.

## 2. Microbial Ecology along the Pig Gastrointestinal Tract

### 2.1. Microbial Composition and Diversity

The pig GIT harbors diverse and dynamic microbial ecosystems consisting of bacteria, archaea, fungi and viruses [39]. Bacteria with cell densities ranging from 10^10^–10^12^ cells per gram colon contents and feces [40] comprise most of the GIT microbiota. According to a recent meta-analysis, the bacterial microbiota of pig GIT encompasses up to 35 different bacterial phyla, 880 genera, and 25,000 operational taxonomic units (OTUs) [14]. *Prevotella* spp. are present throughout the GIT and their abundance, physiological role within the gut, and influence on the host phenotype are constantly influenced by the interaction network structure existing amongst the gut microbial community [12,17]. Thus, to understand the role of *Prevotella* in modulating host phenotype, here, we briefly review the bacterial microbiota present within the pig GIT.

Discrete bacterial communities are present in the different microhabitats along the GIT, with bacterial density, community diversity and evenness increasing from the proximal to distal GIT [14,41,42]. Similar to the pattern where bacterial distribution differs by the longitudinal axis (proximal to distal), bacterial community structure and diversity also differ from lumen to mucosa [15]. As such, the mucosal bacterial community has been found to be significantly richer than that of the lumen in both the ileum and the cecum [14,15].

*Firmicutes*, *Bacteroidetes* and *Proteobacteria* are the most predominant phyla found in the GIT [12,41,43]. *Firmicutes* remains as the most predominant phylum in both upper and lower GIT [44]. The second most abundant phylum in the upper GIT is *Proteobacteria*; however, its abundance decreases in the lower GIT where strict anaerobic *Bacteroidetes* substitutes it [14,15]. Most of the core genera identified within the swine GIT are *Clostridium*, *Blautia*, *Lactobacillus*, *Prevotella*, *Ruminococcus*, and *Roseburia*, RC9 gut group, and *Subdoligranulum* [14,15].

### 2.2. Factors Shaping the Bacterial Microbiota in the Pig Gastrointestinal Tract

**Figure 1:**
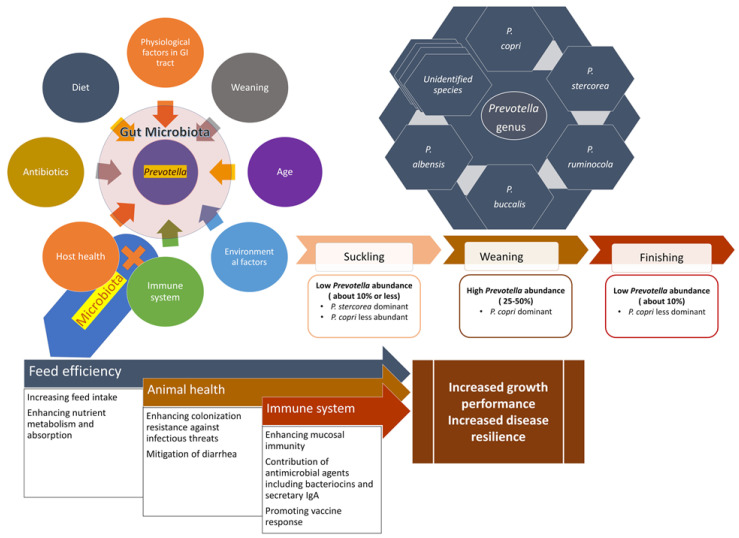
Schematic overview of the factors influencing the microbiota in gastrointestinal tract (GIT) of pig and the changes in relative abundance of *Prevotella* at different stage of pig growth, as well a hypothetical roles of *Prevotrella* spp. in mediating animal performance and health. The relative abundance of *Prevotella* spp. is influenced by many factors that shape gut microbiota. Under the symbiotic relationship with the host, gut microbiota mediates feed efficiency, immune system development, and host health in pigs. As a principal genus of the gut microbiota, *Prevotella* spp. are major contributors to host physiology. There are more than a dozen different species within the genus *Prevotella* present in the pig GIT, and their abundance changes with growth stage.

Different physiological conditions along the GIT account for discrete microbial community structure and composition (Figure 1). The pH in the stomach is significantly lower than the rest of the GIT; thus, the more acid-tolerant bacteria species such as *Lactobacillus* are more abundant [45]. Another physiological factor that may influence the microbial distribution along the GIT is bile [46]. Since bile has detergent and antimicrobial properties, the presence of bile acids in the small intestine favors bacteria that are bile tolerant [46,47,48]. In addition, bacterial colonization in the GIT is also influenced by the mucus layer covering the intestinal epithelium. The mucus layer serves as a physical barrier to keep pathobionts in the GI lumen and acts as a substrate for bacteria, like *Prevotella*, that are able to degrade mucin [49,50,51]. Lastly, the presence of secreted immunoglobulin A (IgA) in the mucosa plays a critical and non-redundant role in monitoring the composition of microbiota [52]. Secretory IgA-deficient humans have been reported to exhibit reduced gut microbial diversity with significant shifts in the relative abundances of specific microbial taxa compared to matched controls [53]. 

In addition to the physiological conditions specific to each of the GIT sections, the overall community structure and diversity of pig gut microbiota are shaped by other factors such as age, diet, and management practices. Longitudinal studies revealed that the pig gut microbiota undergoes significant changes in terms of community structure and composition from birth to market [12,54]. At the neonatal stage, the small intestinal microbiota is dominated by *Lactobacillus*, *Escherichia/Shigella*, and *Bacteroides*, while large intestinal microbiota is dominated by *Prevotella* [55]. However, upon weaning, both small and large intestines are dominated by *Prevotella* [55]. In comparison to large intestine, *Prevotella* is not as dominant in the small intestine [14]. The primary factor that can induce significant changes in the gut microbiota from birth to market across all different growth stages is diet, accounting for up to 34% of variations observed in pig gut microbiota [12]. Besides diet, management practices such as antibiotic treatment and housing are other important factors influencing the pig gut microbiota structure and diversity [56]. Antibiotics are commonly used in pig production to treat or prevent diseases and to improve feed efficiency [57]. However, increasing evidence suggest that the use of antibiotics may disrupt the GIT microbiota structure and function by eliminating the susceptible microbial population and leaving behind resistant strains that can continue to propagate and may be implicated in the development of drug-resistant infections [15,56]. The environment the pigs live in may also have an impact on the pig gut microbiota. For example, pigs raised in complex straw-based housing environment harbor a significantly distinct gut microbiota compared to those raised under simple-slatted housing environment in terms of community structure and composition [58].

Due to the numerous factors that shape the pig gut microbiota, the symbiotic relationship between the gut microbiota and the host is often disrupted, resulting in compromised host health and metabolism [3]. Thus, restoring such homeostasis through the manipulation of the gut microbiota holds great potential to improve pig health. The results of the FMT-based studies have suggested that modulation of the gut microbiota using FMT may enhance growth performance and disease resilience in pigs [11]. Of note, one of the significant changes observed in pig gut microbial composition in response to FMT is a significant increase in *Prevotella* [59], indicating that this genus may be involved in FMT-mediated changes in host phenotype in recipient animals.

## 3. *Prevotella* within the Gut Microbial Community of Pigs

### 3.1. Taxonomy and a Brief History of the Genus Prevotella

The genus *Prevotella* was created in 1990 by Shah and Collins [60] after evidence warranted reclassification from the genus *Bacteroides*. Under the new genus *Prevotella*, named after French microbiologist A.R. Prevot, 16 Gram-negative obligate anaerobic species of *Prevotella* were classified. The species that were transferred to the new genus *Prevotella* were quite distinct in terms of ecological, biological, and chemical characteristics from those of the genus *Bacteroides*. For example, these *Prevotella* species were first obtained from the oral cavity, upper respiratory tract, and urogenital tract, and they were deemed moderately fermentative, sensitive to bile salts, and lacked enzymes such as glucose-6-phosphate dehydrogenase (G6PDH) [60]. In contrast *Bacteroides* species, mainly isolated from the GIT, were fermentative, tolerant to bile salts, and possessed G6PDH [60].

Currently, there are approximately 51 validated species belonging to the genus *Prevotella* [61] (Table 1). These species are known to colonize throughout the host primarily in the oral cavity, upper respiratory tract, urogenital tract, and the GIT [61] (Table 1). The type of species within the *Prevotella* genus, *P. melaninogenica*, was initially isolated from the human oral cavity [60]. Of the species not initially isolated from humans, *P. albensis*, *P. brevis*, *P. bryantii* and *P. ruminicola* were all isolated from ruminants; *P. pectinovora* from pig feces; *P. falsenii* from the oral cavity of monkeys; and *P. paludivivens* from plant residue and rice roots [62,63]. Recently, three novel species, *P. muris*, *P. rodentium*, and *P. intestinalis*, were isolated from mouse colonic content [64]. Interestingly, 16S rRNA gene sequencing results of samples derived from the gut of moths and marine deep sediments have been shown to have 98.8 and 99.3% sequence identity to *P. copri* and *P. tannerae*, respectively [62]. Given the presence of *Prevotella* species in diverse animal species, it is likely that the genus will continue to expand as more environments are explored.

### 3.2. Presence and Abundance of Prevotella along the Pig Gastrointestinal Tract

Overall, *Prevotella* is the most predominant genus present in the GIT of pigs [14,44,55]. According to a meta-analysis performed on 20 publicly available data sets from high-throughput 16S rRNA gene sequencing studies of the pig gut microbiota, *Prevotella* was identified as the most predominant genus within the GIT of pigs, with a mean relative abundance of 17.3% [14]. This meta-analysis also identified that *Prevotella* was detected with high frequency (>97%) in samples that originated from gastric mucosa, duodenum, duodenal mucosa, jejunal mucosa, ileal mucosa, cecal mucosa, colon, colonic mucosa, and feces. Another longitudinal study showed that *Prevotella* was identified as the most predominant genus in the cecum, colon and rectum of pigs on days 7, 21, 28, 35, 120, and 180 of age [55]. Collectively, these studies highlight that the genus *Prevotella* is an important member of both the upper and lower GI microbiotas in pigs. Although the specific functional properties of *Prevotella* spp. along the different GIT sections remain to be defined, it is likely that *Prevotella* spp. are functionally different between upper and lower GIT of pigs. The abundance of *Prevotella* spp. may also be different among the luminal and mucosal bacterial communities at some GI locations. Looft et al. [15] observed that mucosal microbiota harbored significantly greater abundance of *Prevotella* compared to luminal microbiota in the same site of small intestine (ileum, 9.3% vs. 0.01%) in adult pigs. In the large intestine, however, *Prevotella* was found to be equally abundant in both luminal and mucosal microbiota compartments [15]. Of note, most data on *Prevotella* abundance are proportional, rather than quantitative, which sometimes limits interpretation.

The abundance of *Prevotella* in the pig GIT fluctuates with growth stages. *Prevotella* spp. are less abundant during the suckling and nursery stages but become more dominant during the growing and finishing stages after weaning [12,19,74,75,76]. In 2018, Guevarra et al. [74] evaluated the bacterial communities in fecal samples collected from 10 healthy piglets just prior to weaning (21 d of age) and one week after weaning (28 d of age) using 16S rRNA gene sequencing, and they found that *Prevotella* increased from 12.9% to 57% over that period. In addition, Mach et al. [19] found that the relative abundance of *Prevotella* was 1% in suckling piglets (d 14), 27% within first week post weaning (d 36), and continued to increase in the subsequent 5 weeks (31%, 41%, 43% on days 48, 60, and 70, respectively). However, the fecal microbiota of sows harbored only about 8% *Prevotella*, indicating that there may be a point where relative abundance begins to decline.

Switching from milk-based to complex plant-based diets at weaning results in an abrupt change in the carbohydrate composition of the pig diet [77]. As a result, the microbial composition shifts towards microbes that can metabolize such substrates [77]. Metagenomic studies have shown significant alterations in the functional capacity of gut microbiota following the ingestion of complex plant-derived glycans, with greater abundance of genes involved in xylose utilization, mannose metabolism, and L-rhamnose utilization [74,78,79]. Species within the genus *Prevotella* can breakdown the plant cell wall through enzymes such as xylanases, mannanases and β-glucanases [19,80]. Thus, the dramatic changes in *Prevotella* abundance upon weaning is likely an important contributor to alteration of the overall function of gut microbiota.

### 3.3. Different Prevotella Species Present in the Pig Gastrointestinal Tract 

Among the *Prevotella* species listed in Table 1, about 50% of these species have been reported in the pig GIT. The most frequently identified *Prevotella* species in samples from the pig GIT are *P. copri*, *P. stercorea* and *P. ruminicola* [7,12,19,81,82]. Nursing piglets may harbor more *P. stercorea* in the first 3 weeks of life while it depletes as the piglets grow [12]. *P. copri*, however, is present during the nursing period at low abundances, but increases drastically upon weaning, and continues to be more abundant in piglets at the nursery and growing stages while decreasing in the finishing stage [12]. Due to the fact that most of the studies characterizing the gut microbiota in pigs are based on 16S rRNA gene sequencing, which limits the identification of bacteria at the species level, there is inadequate information with respect to the complete survey of the different *Prevotella* species within the GIT. This may be informed by both metagenome assembled genomes as well as whole genome sequencing of new isolates.

## 4. *Prevotella* in Feed Efficiency and Growth Performance

Regulation of feed intake and feeding behavior by the gut microbiota has recently been identified in pigs (Figure 1 and Table 2). Certain taxa including *Prevotella* spp. have been linked to microbiome-mediated feed intake in pigs. For example, results from 16S rRNA gene amplicon sequencing of fecal samples obtained from 280 commercial Duroc pigs revealed that the pigs that harbored a *Prevotella*-predominant enterotype had significantly higher average daily feed intake (ADFI) compared to the pigs characterized by the *Treponema* enterotype [18]. In this study, among the 18 OTUs that exhibited strong positive association with ADFI, 67% were assigned to the genus *Prevotella*. Moreover, the network analysis performed on the ADFI-associated OTUs showed that *Prevotella* was a hub bacterium in the co-abundance network. Thus, it is possible that *Prevotella* spp. may promote feed intake in pigs, warranting further research into the manipulation of gut *Prevotella* to enhance feed intake and thereby promote growth performance. There is currently no proposed or demonstrated mechanism through which *Prevotella* increases feed intake, and *Prevotella* enrichment may be a product of increased feed intake rather than a driver of feed intake.

Other studies suggest that *Prevotella*-enriched gut microbiota may enhance growth performance in pigs (Table 2). Mach et al. [19] reported that, after weaning, the gut microbiota of healthy piglets (n = 31) were clustered into two different enterotypes (unclassified *Ruminococcaceae* or *Prevotella-*enriched enterotypes). Although the piglets in the *Prevotella* cluster exhibited lower growth rates during suckling, these animals showed higher body weight and average daily gain compared to animals belonging to the *Ruminococcaceae* cluster. The authors argued that the increased growth performance in *Prevotella*-enriched animals post weaning might be due to the ability of *Prevotella* to ferment complex dietary polysaccharides, which ultimately increases energy harvest, though not measured in the study. In addition, Ramayo-Caldas et al. [17] identified a gut microbial ecosystem structure linked with growth traits in pigs. They constructed two networks at the genus and OTU levels based on 16S rRNA sequencing data from the fecal samples of a cohort of 518 healthy 60-day old pigs. Within network interactions, there was a strong co-exclusion between *Prevotella* and *Ruminococcus* genera. The piglet samples were clustered into two enterotype-like groups, which were dominated by either *Ruminococcus* and *Treponema* (PEA), or *Prevotella* and *Mitsuokella* (PEB). The animals that clustered into the PEB group had significantly greater body weight at 60 days of age and average daily gain compared to the PEA group, indicating a positive link between *Prevotella* predominant gut microbiota and favorable growth traits in pigs. This is further supported by Kiros et al. [83], who observed a positive correlation between piglet average daily gain and the abundance of *Prevotella* in piglets that received a diet supplemented with high yeast (*Saccharomyces cerevisiae*). Wang et al. [12] also identified *Prevotella* spp. (*Prevotella copri* and several unclassified *Prevotella* OTUs) as among the top 50 growth performance-associated taxa at suckling, nursing, growing, and finishing stages.

In contrast, there are some studies indicating that the abundance of *Prevotella* spp. is inversely correlated with feed efficiency in pigs. Tan et al. [79] observed a distinctive microbial structure and composition of cecal microbiota between low and high feed efficient (FE) pigs. Although *Prevotella* was the most enriched genus in both groups, low FE pigs harbored significantly higher abundance of *Prevotella* sp. CAG:604 taxa compared to high FE pigs. The authors suggested that the species *Prevotella* sp. CAG:604 might be a potential biomarker for distinguishing between the cecum microbiota of high and low FE groups. Thus, certain *Prevotella* species in the GIT may have adverse effects on the growth traits of pigs. Notably, these effects may be species or strain specific rather than common across all *Prevotella* spp. Another study also reported that the group of pigs that had low feed conversion ratio (FCRs) were colonized by greater abundances of taxa within the *Prevotellaceae* family in the ileum than high FCR animals [84]. In addition, Unno et al. [85] observed similar negative correlations between host weight and the abundance of *Prevotellaceae* family within the GIT. Furthermore, Yang et al. [82] evaluated the association of fecal microbiota with feed efficiency in 280 commercial Duroc pigs and reported that the pigs that clustered into the *Treponema*-dominant enterotype tended to have lower residual feed intake (RFI) values than those clustered into the *Prevotella*-dominant enterotype.

Overall, the existing studies showing the positive associations of *Prevotella* with feed efficiency and growth performance in pigs outnumber those indicating a negative association with these animal traits (Table 2). However, these contradictory studies highlight the need to investigate the role of specific *Prevotella* spp. in feed efficiency in pigs. We can speculate that the specific *Prevotella* spp. or strains associated with positive and negative growth outcomes differ, however, mechanistic studies with controlled *Prevotella* colonization are needed to demonstrate a causal role in altering growth performance. 

## 5. *Prevotella* and Diarrhea in Pigs

The gut microbiota can mediate the development of diarrhea in pigs, and it has been shown that animals that harbor higher *Prevotella* may have better protection against diarrhea (Table 2). Dou et al. [10] observed that healthy piglets, which were weaned in poor housing conditions to challenge their susceptibility to diarrhea, harbored gut microbiota enriched with *Prevotellaceae*, *Lachnospiraceae*, *Ruminococcaceae* and *Lactobacillaceae* compared to diarrheic piglets. Sun et al. [86] also reported that non-diarrheic piglets harbored significantly greater abundance of gut *Prevotella* compared to diarrheic piglets. Co-correlation network analysis revealed that *Prevotellacea* UCG-003 was the key bacterium in the non-diarrheic microbiota of piglets, whereas the genus *Escherichia-Shigella* was the core component of diarrheic microbiota. The inverse association of the abundance of *Prevotella* with the incidence of diarrhea in pigs is further supported by a study in which the higher abundances of *Prevotella* have also been reported in piglets affected by diarrhea. Neonatal piglets with diarrhea exhibited significant enrichment of *Prevotella* [82]. The increase in *Prevotella* abundance was correlated with the depletion of *E. coli*, and *Lactobacillus*, *Enterococcus*, *Streptococcus* and *Clostridium* in diarrheic piglets. Thus, the authors speculated, based also on observed changes in microbial functional gene profiles in diarrheic piglets, that dysbiosis of the gut microbiota may create an environment in the GIT that favors the proliferation of *Prevotella* in diarrheic conditions [82]. Similarly, another study that evaluated the longitudinal development of the gut microbiota in healthy and diarrheic piglets induced by age related dietary changes showed that *Prevotella* was in greater relative abundance in diarrheic piglets than in healthy piglets receiving early supplementary creep feed or sows’ milk [88]. The weak negative correlation between *Prevotella* and *Escherichia* observed with diarrheic piglets suggested that the disruption of the competitive relationship between *Prevotella* and *Escherichia* may have implications in predisposing piglets to diarrhea [88].

While the role of *Prevotella* in pathogenesis of pig diarrhea warrants further research, the interactions of *Prevotella* with other commensals and the presence of different *Prevotella* species at different growth stages may have implications in dictating the role of *Prevotella* to be a diarrhea preventative or promotive. The *Prevotella* genus is certainly not exclusively beneficial, as many *Prevotella* spp. (Table 1) have been associated with infections within the oral cavity, lower respiratory tract, central nervous system, abdominal and female genital tract [61,66]. Thus, characterization of *Prevotella* at species or even strain level in gut microbiota may enhance conclusive understanding of the relationship between *Prevotella* and incidence of diarrhea in pigs. Again, controlled studies with exposures to specific *Prevotella* species or strains will be required to demonstrate a causal role.

## 6. *Prevotella* and the Intestinal Immune System

The intestinal microbiota is important for the development and modulation of the gut mucosal immune system in pigs [89,90]. *Prevotella*, being one of the most predominant genera among the intestinal bacteria in both pre-and post-weaned pigs, may contribute to the microbiota-induced mucosal immune development. Although there is limited information with respect to the impact of *Prevotella* on mucosal immunity in pigs, existing evidence derived from humans and other mammals suggests that *Prevotella* spp. may contribute to the maturation of mucosal immunity in several ways [91,92]. One possible way might be through its association with short chain fatty acid (SCFA)-mediated mucosal immune homeostasis. *Prevotella* produces acetate as an end product of anaerobic microbial fermentation in the intestine [64,93]. Acetate is utilized by other commensals such as *Roseburia* and *Faecalibacterium* to produce butyrate [91]. Butyrate is an important microbial-derived SCFA that has been shown to benefit intestinal development and maintenance, and immune defense functions in mammalian species [92]. Butyrate is a primary energy source of colonocytes, and it is also involved in mucosal immune development and priming the mucosal defense against infectious threats [94]. Microbial-derived butyrate has been shown to promote epithelial barrier function through IL-10 receptor-dependent repression of claudin-2 [95]. Studies have shown that the capacity of microbial SCFA production varies in *Prevotella*- versus *Bacteroides*-dominated gut microbiota [95]. From the same carbohydrate substrates, *Prevotella*-dominated microbiota produced different profiles of SCFAs, and produced 2–3 times more propionate than the *Bacteroides*-dominated microbiota [95].

The *Prevotella*-dominant enterotype has also been associated with higher production of secretory IgA in adult pigs compared to the *Ruminococcaceae* enterotype [19]. The gut secretes significant amounts of IgA antibody that serves as the first line of innate defense against invading pathogens [96]. Secretory IgA also facilitates effective communication between the commensal microbiota and the immune system by selective presentation of commensal species to tolerogenic dendritic cells, thereby restricting systemic adaptive response to resident commensals [52]. It has been demonstrated that *Bacteroides fragilis* relies on IgA response to occupy a defined mucosal niche that mediates stable colonization of the gut through exclusion of exogenous competitors [97]. The fact that *Prevotella* abundance associates with elevated fecal IgA as well as improved growth performance may support the possibility that increased IgA is stimulated by *Prevotella* to maintain a symbiotic relationship with the host. It is also possible that *Prevotella* does not induce an IgA response and simply benefits from elevated levels of secretory IgA. Studies exploring the detailed and mechanistic nature of *Prevotella’s* association with an IgA response are warranted, as elevated IgA correlates with performance [19].

There are also some reports showing that piglets that harbor *Prevotella*-dominated gut microbiota may be more susceptible to develop chronic inflammatory disease (e.g., colitis). For example, Xiao et al. [98] reported that the fecal microbiota of piglets that were less resilient against dextran sulphate sodium-induced acute colitis contained significantly greater abundance of *Prevotella* (35% vs. 24%) compared to the more resilient ones. *Prevotella* spp. have been reported to be involved in the pathogenesis of ulcerative colitis in humans [99], although not consistently. In addition, there is a growing body of evidence in humans suggesting a link between the increase in *Prevotella* spp. at the intestinal mucosal site to localized and systemic disease [30,64]. Iljazovic et al. [64] investigated the potential causal role of *P. intestinalis* nov. sp. strain in intestinal dysbiosis and inflammation. Colonization with *P. intestinalis* decreased IL-18 production, which exacerbated colonic inflammation in immunocompetent mice [64]. Strikingly, when recombinant IL-18 was administered back to these mice, colitis symptoms, including inflammation, were decreased [64]. This study highlights the need for mechanistic experiments to determine the causal role(s) *Prevotella* spp. play in the immune system, not only in mice, but in other models. Furthermore, multiple lines of evidence derived from human and mice studies suggest the potential role of *Prevotella* dominant intestinal microbiota in the development of autoimmune diseases such as Rheumatoid arthritis [72,100]. Thus, it is important to investigate the role of *Prevotella* spp. in the development of intestinal inflammatory disorders in pigs.

It has been also suggested that an increase in *Prevotella* spp. in the intestinal tract of pigs may have a negative impact on the integrity of the intestinal mucus layer, and increased abundance of *Prevotella* spp. has been associated with penetrable mucus [101]. Some *Prevotella* spp. can degrade sulfated mucin glycans and thereby may affect the mucus layer [51,102]. In addition, *Prevotella copri* has been identified to act as modulator of infection caused by foodborne pathogen *Listeria monocytogenes* [103]. Precolonization of germ-free mice with *Prevotella copri* strain resulted in a significantly thinner mucus layer and higher degree of intestinal inflammation in mice following *Listeria monocytogenes* inoculation compared to those preinoculated with other commensal bacteria (*Bacteroides thetaiotaomicron*), suggesting that *Prevotella copri* may impair intestinal mucus barrier function, and therefore making the intestinal epithelial cells and local inflammation system more vulnerable to pathogen invasion [103]. However, the *P. salivae* strain did not exhibit any role in exacerbation of intestinal infection associated with *Listeria monocytogenes* [103]. To our knowledge, no study has investigated the role of *Prevotella* in mucus defense in pigs, but it is an area of research that future studies should investigate.

## 7. *Prevotella* and Vaccine Response

The potential role of gut microbiota in the regulation of host immune responses to vaccines has garnered more attention [104,105,106]. Various models have been used to study dysbiotic microbiota and vaccine response, such as germfree mice, antibiotic treated conventional mice [107], and gnotobiotic piglets transplanted with dysbiotic microbiota [108]. Both microbial composition and community diversity has been suggested to influence vaccine response [109]. While the underlying mechanisms by which gut microbiota influences vaccine response remains elusive, some evidence indicates a potential link between an increase in intestinal *Prevotella* and enhanced vaccine response. For example, a positive correlation was observed between *Prevotella* abundance and vaccine responsiveness in 28-day old piglets at the time of vaccination against *Mycoplasma hyopneumoniae* [110]. A parallel study performed in France on a separate population of pigs found the same association of *Prevotella* with elevated antibody titers following vaccination against *M. hyopneumoniae* [76]. According to the blood transcriptome analysis performed on samples obtained 2 days post-vaccination, biological processes associated with cell recruitment were more activated in high vaccine responders that harbored higher abundances of *Prevotella* [110]. Lipopolysaccharide originating from *P. intermedia* has been shown to act as an immunological adjuvant in mice vaccinated against hepatitis B virus [111]. While the data to date are correlational, it is tempting to speculate that increased *Prevotella* abundance may support a more robust response to vaccines through an adjuvant role.

## 8. *Prevotella* and Bacterial Interactions

*Prevotella* may also contribute to mucosal defense by direct or indirectly affecting antimicrobial peptide production in GIT. Strains within *P. intermedia* [112] and *P. nigrescens* [113] have been shown to produce bacteriocins. Most commensal bacteria residing within GIT have the ability to produce bacteriocins [114]. *Prevotella*, being highly interactive with other bacterial species in the gut, may also influence the capacity of bacteriocin production by other individual commensals. Multiple lines of evidence suggest that there is relatively strong species–species interaction network that exists between *Prevotella* spp. and other bacteria in pig GIT. Network analysis identified that the genus *Prevotella* exhibited close interconnectivity with many other taxa in both cooperative and competitive manners [18]. Wang et al. [12] identified *P. corpi* as key taxon that connects lactation and growing stage-specific bacterial clusters to one another, indicating that *P. copri* has close interactions with other gut microbial community members in both pre- and-post weaning pigs. Highly connected taxa have recently been termed as keystone taxa [20]. The keystone taxa drive the microbiome structure and functioning irrespective of their abundance across space and time [20]. Furthermore, microbial interactions shape host physiology, which was demonstrated by the fact that higher-order bacterial interactions accounted for a 28% increase in the life span of fruit flies [115]. Thus, *Prevotella*, being a highly connected genus within the GIT microbial community, may influence the overall metabolic function of gut microbiota. In addition to the network analysis, in vivo colonization studies also indicated that *Prevotella* interact with other beneficial and pathogenic bacteria within the gut. For example, a probiotic-based study suggested that species within *Prevotella* and *Lactobacillus* may have close interactions and thereby mutually support one another in the pig gut [74]. Interaction between gut *P. copri* and pathogenic bacteria *Listeria monocytogenes* has also recently been demonstrated in an infectious mouse model [103]. Interaction of *Prevotella* with other bacteria of the gut microbiota could be an important factor that influences how *Prevotella* behaves in different gut ecosystems and its interactions with the host.

## 9. Challenges and Future Opportunities Associated with Harnessing *Prevotella* to Improve Pig Health

### 9.1. Challenges

The characterization of *Prevotella* spp. in the pig GIT has largely relied on culture independent methods, particularly 16S rRNA gene-based high throughput sequencing [14]. Although culture-independent methods enable researchers to study the abundance of *Prevotella* spp. in different GIT locations and their associations with animal performance and health outcomes at different growth stages, the information obtained by these methods is primarily limited to genus level. To date, the vast majority of studies are limited to correlations between the abundance of *Prevotella* and pig production and health, making it challenging to identify the role of *Prevotella* in defining host phenotype. The diversity of *Prevotella* species found in the GIT of pigs (e.g., *P. copri*, *P. stercorea*, *P. ruminicula* and *P. oulorum*) [12,73] and the known variation from symbiont to pathogen within the species indicate that genus level classification is insufficient. Even within a *Prevotella* species, multiple OTUs get classified as the same species [73]. For example, a study presented that 68 and 17 different OTUs were annotated to *P. copri* and *P. stercorea*, respectively [73]. Moreover, a recent comparative genomic analysis performed on more than 1000 *P. copri* genomes originating from the human gut represented by multiple host-geographies, disease and lifestyles revealed that *P. copri* is not a monotypic species but may be comprised of four different clades [116], all of which have the potential to reside solely or in combination, despite their distinct genomic diversity. Given the number of *P. copri* OTUs identified in many studies, it is likely that a *P. copri* complex also exists in the pig gut, however, this has not been explored. Given that antimicrobial and immunomodulation characteristics of bacteria are species and often strain specific [117], the functional features of *Prevotella* in the gut are also likely highly specific (Figure 1), which prompts the need for characterizing the role of most common *Prevotella* species in pigs (e.g., *P. copri* and *P. stercorea*) using metagenomic and culture dependent approaches. Apart from the application of whole genome sequencing for species and strain-level microbiome analysis [118,119], high throughput sequencing of the full 16S rRNA gene has shown the potential to provide taxonomic resolution of bacterial communities at species and strain level [120]. Thus, using the Pacific Biosciences (PacBio) RS II platform [120] to sequence the full 16S rRNA gene of the bacterial microbiota in pig GIT should be considered to identify the link between the host phenotype and specific *Prevotella* species or strains.

The strict obligate anaerobic nature of *Prevotella* spp. hinders their successful growth on non-selective growth media under standard anaerobic growth conditions. There is relatively limited information available with respect to culturing *Prevotella* species originating from the intestinal tract of pigs. Ghimire et al. [121] was able to culture and isolate *P. copri* and *P. stercorea* strains from the human fecal samples of healthy adults using a modified brain heart infusion (BHI) agar plate incubated at 37 °C for 48 h under the following anaerobic gas condition: 85% N_2_, 10% CO_2_ and 5% H_2_ gas. Under the same media and growth conditions, these authors were able to isolate *Prevotella* from cecum and colon samples of adult Tamworth pigs. Having said this, the isolation frequency of *Prevotella* was relatively low (1.4% of 500 colonies were identified as *Prevotella*), considering the relative abundance of *Prevotella* genus constituted 38% of the total microbiota in the donor pigs [122]. Thus, developing highly selective culture media and optimizing growth conditions for isolation of *Prevotella* species are needed.

In 2007, Hayashi et al. [123] first isolated *P. copri* and *P. stercorea* from feces of a healthy Japanese male using Eggerth Gagnon agar supplemented with 5% (*v*/*v*) horse blood. These plates were incubated for 48 h at 37 °C in an atmosphere containing 100% CO_2._ According to this study, the growth of *Prevotella* spp. may require an anaerobic atmosphere with higher concentrations of 10% CO_2_ or higher. As such, a recent study identified that *P. copri* depends more heavily on the addition of CO_2_ or bicarbonate for biomass formation compared to *Bacteroides* spp. [93]. With this knowledge, our research team has been working to culture *Prevotella* species originating from the pig intestine. Using peptone yeast glucose (PYG, modified from the original media developed by Varel and Bryant [124]) media under anaerobic conditions containing 20% CO_2_ and 80% N_2_ mixed gas, *P. copri* was successfully isolated from conventional sow feces. Given that various *Prevotella* species are present within the GIT of pigs, developing culture media and identifying optimal growth conditions that support the growth of multiple *Prevotella* species should be the focus of future research. 

### 9.2. Future Opportunities

The increasing applications of meta-omics including shotgun metagenomics, metatranscriptomics, metaproteomics, and metabolomics in functional and translational microbiome research enable comprehensive characterization of the composition (at strain level), functional, and metabolic activities of complex microbiomes [125]. With continuing decline in the cost of these meta-omic approaches and rapid advances in data integration, there will be an increased application of the integrated meta-omics in studying pig gut microbiota. As a result, uncovering the role of gut microbiota in pig health and productivity will advance, ultimately providing a basis for microbiome-targeted strategies to be employed. Developing *Prevotella* spp.-based next-generation probiotics might be a logical first step to manipulate the *Prevotella* species of interest in pig gut and thereby improve feed efficiency and gut health. While there are no probiotics *Prevotella* spp. currently available for pigs, *Prevotella bryantii* strains (25A and 3G5) have been used as probiotics in early-lactation dairy cows and sheep to improve ruminal fermentation products and milk fat concentration [126,127]. *Prevotella copri*, the most predominant species in GIT of adult pigs, could be a good candidate for the development as a next-generation probiotic. Thus, the role of *P. copri* originating from the pig in fermentation dynamics and interaction with the host should be characterized in vitro and in vivo using cell culture, chemostat gut model systems and controlled colonization models including integrated multi-omics analysis.

## 10. Conclusions

Although much progress has been made in understanding the role of gut microbiota in pig health and productivity in recent years, this knowledge is still largely limited to the association of overall community diversity and composition with certain host phenotypes. Thus, identifying causational roles of individual members of the gut microbiota on host phenotype is essential to create strategies targeted at improving host health and productivity. Therefore, further investigation on the causational role of the species within *Prevotella* in regard to feed efficiency, immune response, and disease resilience in pigs will not only elucidate the principles that govern the gut microbiota and host interactions, but also provide important information to design microbiome-mediated strategies to achieve desired host phenotypes. In order to explicate the causal relationship between *Prevotella* spp. and the host, the application of controlled colonization models coupled with meta-omic techniques, and improved culture techniques are required.

## Figures and Tables

**Table 1:** Summary of the species in the genus *Prevotella* ^1^.

*Prevotella* Species	Associated with Human Infections ^2^	Presence in Pig GIT ^3^	Comment	References
*P. albensis*	-	Yes	Initially isolated from ruminants	[65]
*P. amnii*	-	-	-	[61]
*P. baronia*	Yes	Yes	-	[60]
*P. bergensis*	Yes	Yes	-	[61]
*P. bivia*	Yes	Yes	-	[61]
*P. brevis*	-	-	Initially isolated from ruminants	[65]
*P. bryantii*	-	Yes	Initially isolated from ruminants	[65]
*P. buccae*	Yes	Yes	-	[61]
*P. buccalis*	Yes	Yes	-	[61]
*P. conceptionensis*	Yes	-	-	[66]
*P. coporis*	Yes	-	-	[61]
*P. copri*	Yes	Yes	Highly abundant pig gut after weaning	[61]
*P. dentalis*	Yes	Yes	-	[61]
*P. denticola*	Yes	Yes	-	[61]
*P. disiens*	Yes	Yes	-	[61]
*P. enoeca*	Yes	Yes	-	[60]
*P. heparinolytica*	Yes	-	-	[61]
*P. histicola*	Yes	-	-	[61]
*P. falsenii*	-	-	Initially isolated from monkey	[67]
*P. fusca*	-	Yes	Human oral cavity	[68]
*P. intermedia*	Yes	-	-	[61]
*P. intestinalis*	-	-	Mouse colonic content	[64]
*P. loescheii*	Yes	-	-	[61]
*P. massilliensis*	-	-	-	[60]
*P. maculosa*	-	Yes	-	[61]
*P. marshii*	-	-	-	[61]
*P. melaninogenica*	Yes	Yes	-	[60]
*P. micans*	-	Yes	-	[61]
*P. multiformis*	-	-	-	[60]
*P. multisaccharivorax*	-	-	-	[61]
*P. muris*	-	-	Mouse colonic content	[64]
*P. nanceiensis*	Yes	-	-	[66]
*P. nigreceiensis*	-	-	-	[61]
*P. nigrescens*	Yes	Yes	-	[60]
*P. oralis*	Yes	Yes	-	[60]
*P. oris*	Yes	Yes	-	[60]
*P. oulora*	-	-	-	[69]
*P. oulorum*	Yes	Yes	-	[69]
*P. pallens*	Yes	-	-	[61]
*P. pectinovora*	-	-	Initially isolated from pig feces	[63]
*P. pleuritidis*	-	-	-	[61]
*P. rodentium*	-	-	Mouse colonic content	[64]
*P. ruminicola*	Yes	-	Initially isolated from ruminants	[65]
*P. saccharolytica*		Yes	Human oral cavity	[70]
*P. salivae*	Yes	Yes	-	[61]
*P. scopos*	-	-	Human oral cavity	[68]
*P. shahii*	-	Yes	-	[61]
*P. stercorea*	-	Yes	More abundant in suckling piglet gut	[61]
*P. tannerae*	-	-	-	[61]
*P. timonensis*	Yes	-	-	[61]
*P. veroralis*	Yes	-	-	[61]
*P. zoogleoformans*	Yes	-	-	[60]

^1^ This list does not contain all the *Prevotella* species identified up to date, but it covers the list of species commonly reported from human, ruminants and pigs. ^2^ The association with human infections was determined based on the information reported by Jousimies-Somer and Summanen [71], Ulger Toprak et al. [66] and Maeda [72]. ^3^ Based on the operational taxanomic units (OTUs) assigned to (>97% sequence similarity) different *Prevotella* species. These data were derived from the pyrosequencing of 16S rRNA genes from stomach, ileum and colon samples of weaned piglets [73].

**Table 2:** The summary of studies (n = 11) showing the potential association of gut *Prevotella* with growth performance (positive) and diarrhea (negative) in pigs (determined by high throughput sequencing techniques).

Study Categories	Animals	Country of Origin	Collected Samples	Point of Sample Collection	Samples Processed for	*Prevotella* Abundance	Main Findings	References
*Prevotella* and enterotypes of pig gut microbiota and host performance	A cohort of 953 pigs from a F_6_ population of heterogeneous pig cross	China	Fecal	At the ages of 25, 120 and 240 days, which represented the time of preweaning, mid-stage of fattening and slaughtering (weaned at d 28)	16S rRNA gene sequencing (V3-V4); Illumina MiSeq	Day 25 (preweaning): *Fusobacterium* vs. *Prevotella* dominant enterotypes Days 80, 120 and 240: *Treponema* vs. *Prevotella*-dominant enterotypes	Besides the piglets, even some adult pigs switched putative enterotypes between ages	[16]
						At all sampling time points, *Prevotella* was most abundant and served as one of the two main network hubs	The topological features of phylogenetic cooccurrence networks, including scale, stability and complexity were increased along with the age	
	A total of 575 Large White pigs	France	Fecal	At the age of 60 days (weaned at d 28)	16S rRNA gene sequencing (V3-V4); Roche 454 GS FLX Titanium	*Ruminococcus* and *Treponema* vs. *Prevotella* and *Mitsuokella*-driven enterotype (PEA vs. PEB)	Diversity analysis revealed a significantly higher level of alpha-diversity and richness for PEA than for PEB	[17]
							Animals that clustered with the PEB were on average 850 g heavier and had an extra average daily gain (ADG) of 17.9 g per day than those that clustered with the PEA	
							Showed the link between microbial ecosystems and pig growth traits	
	280 commercial Duroc pigs	China	Fecal	At the age of 140 days (weaned at d 28)	16S rRNA gene sequencing (V4); Illumina MiSeq	*Prevotella* vs. *Treponema*- predominant enterotypes	12 out of the 18 OTUs positively associated with the average daily feed intake (ADFI) were annotated to *Prevotella*, and *Prevotella* was the hub bacteria in the co-abundance network. These results suggest that *Prevotella* might be a keystone bacterial taxon for increasing host feed intake.	[18]
	1039 pigs	USA	Rectal swabs	At weaning (18.6 ± 1.09 days), week 15 (118.2 ± 1.18) days, and off-test (196.4 ± 7.86 days)	16S rRNA gene sequencing (V4); Illumina MiSeq	At weaning: *Prevotella* (6.78%) and the 7th predominant genus; week 15: *Prevotella* (13.1%), and the 1st predominant genus; off-test (at slaughtering): *Prevotella* (6.74%), and the 2nd predominant genus	*Prevotella* dominant enterotype was observed at weaning stage. However, no significant correlations between any enterotypes at weaning and average daily gain were detected	[43]
*Prevotella* and its positive association with growth performance in pigs	18 pigs	USA	Rectal swabs	During lactation (days 0, 11, 20), nursery (d 27, 33, 41, 50, 61), growing (d 76, 90, 104, 116), and finishing (d 130, 146, 159, 174) stages	16S rRNA gene sequencing (V4); Illumina Miseq	Among the top 30 taxa, 11 belong to genus *Prevotella*, the most diverse and dominant genus throughout most of the stages, especially after the introduction of solid feed	*Prevotella* spp. (*Prevotella copri* and several unclassified *Prevotella* OTUs) were identified as one of the top 50 growth performance-associated taxa at lactation, nursing, growing and finishing stages	[12]
	31 healthy piglets	France	Fecal	At the ages of 14, 36, 48, 60 and 70 days (weaned at d 28)	16S rRNA gene sequencing (V3-V4); Roche 454 GS FLX Titanium	After weaning, the microbiota composition coevolved with their hosts towards two different clusters: unclassified *Ruminococcaceae* vs. *Prevotella*	*Prevotella* cluster was positively correlated with luminal secretory IgA concentrations, and body weight	[19]
	A total of 48 piglets (control vs. low vs. high yeast supplemented groups, n = 16)	Canada	Cecum content	At the age of 28 days (at euthanization); body weight measured at 1, 3, 7, 10, 17, 24 and 28 days	16S rRNA gene sequencing (V1-V3); Roche 454 FLX Titanium	Relative abundance of *Prevotella* genus in piglets receiving low or high yeast supplementation was 0.46 and 3.07%, respectively	Partial least squares analysis showed that piglet average daily gain (ADG) was positively correlated with genus *Prevotella* in the high yeast group	[83]
*Prevotella* and its negative association with diarrhea in pigs	20 piglets were weaned in poor housing conditions to challenge their susceptibility to post-weaning	France	Fecal	At the age of 7, 14, 21, 30, 38 and 47 days	16S rRNA gene sequencing (V1-V3); Illumina MiSeq	*Prevotellaceae* families were increased in healthy pigs compared to diarrheic pigs	The higher abundance of *Prevotella* may contribute to allowing healthy pigs better adapt to post-weaning dietary conditions, thereby mitigating the risk of developing diarrhea	[10]
						At the genus level, the higher abundance of Roseburia, *Prevotella* and genera belonging to *Ruminococcaceae* was increased in healthy pigs		
	85 commercial piglets	China	Anal swab	During the lactation (0–19 days old), weaning (20–21 days old), and post-weaning periods (22–40 days)	16S rRNA gene sequencing (V4); Illumina Miseq	*Prevotella* was the one of the 11 genera whose abundance was significantly higher in non-diarrheic piglets compared to diarrheic piglets	*Prevotellacecea* UCG-003 was identified as a key node in non-diarrheic piglets upon co-correlation network analysis	[86]
						The relative abundances of OTUs belonging to *Prevotella*2 and *Prevotella*9 were 0.789% and 0.849% from diarrheic piglets, and 1.787% and 1.692% in the non-diarrheic samples		
	14 piglets from healthy and porcine epidemic diarrhea virus (PEDV) infection-diagnosed group (n = 7)	South Korea	Fecal	Not provided	16S rRNA gene sequencing (V3); Illumina MiSeq	Relative abundance of most commensal bacteria including *Prevotella* and Faecalibacterium) in healthy pigs was decreased following dysbiosis induced by PEDV infection	Reduction of these commensal bacteria including *Prevotella* may have implications in pathogenesis of PVDV-associated diarrhea in pigs	[87]
	51 piglets, and among which 41 piglets were orally challenged with enterotoxigenic *Escherichia coli* (ETEC)	China	Jejunal and fecal	Fresh feces were collected from day 1 to day 5 (post diarrhea infection (PDI)); while cecum jejunal samples were collected at day 6 PDI	16S rRNA gene sequencing (not provided); Illumina MiSeq	Healthy piglets had higher abundance of *Prevotella* in the feces, but lower *Lactococcus* in the jejunum and lower *Escherichia/Shigella* in the feces compared to diarrheal piglets	ETEC-induced diarrhea is associated with the alteration of intestinal microbiota, including lower *Bacteroidetes*: *Firmicutes* ratio and microbiota diversity in the jejunum and feces, and lower *Prevotella* in the feces, but higher percentage of *Lactococcus* in the jejunum and *Escherichia/Shigella* in the feces	[13]
						*Prevotella* (4.2, 1.7 to 0.2%) decreased as the piglets were transient from pre-diarrheic state to diarrheic state		
						Compared to resistant piglets, the diarrheal piglet harbored lower *Prevotella*		
